# Case Report: rare hybrid lesion of a central giant cell granuloma within a juvenile ossifying fibroma

**DOI:** 10.12688/f1000research.19891.1

**Published:** 2019-07-30

**Authors:** Hadeer Rizk Saad, Noura M. Kamal, Hatem W. Amer

**Affiliations:** 1 Department of Oral and Maxillofacial Pathology, Misr International University, Cairo, Egypt; 2 Department of Oral and Maxillofacial Pathology, Cairo University, Giza, Egypt

**Keywords:** Aggressive lesion, Central giant cell granuloma, Fibrous dysplasia, Hybrid lesion, Juvenile ossifying fibroma, Pathology

## Abstract

Central giant cell granuloma (CGCG) is classified by the World Health Organization as a benign bone lesion. It is found anteriorly in the mandible, with most of the cases crossing the midline. In total, 70% of CGCGs are encountered in young females. Fibro-osseous lesions are a group of pathologies that encompass neoplastic, dysplastic and reactive entities. Juvenile ossifying fibroma, which can be further categorized into juvenile trabecular ossifying fibroma (JTOF) and juvenile psammomatoid ossifying fibroma, represents an aggressive neoplastic example of these fibro-osseous lesions. JTOF occurs in children at almost equal ratios in both sexes, affecting the maxilla more than mandible. This study aims to report a peculiar case of a hybrid lesion comprising CGCG and JTOF in the mandible of a nine-year-old female patient. Clinical, radiographic and histopathological findings were assessed. Clinical examination revealed an intraoral swelling extending from the right impacted third molar area to the left first molar area. Computed tomography showed a well-defined multilocular radiolucency with diffuse flecks of radioopacities. Histopathologically, the lesion comprised fibrous connective tissue encompassing numerous multinucleated giant cells and other areas of cell-rich connective tissue stroma containing bands of osteoid matrix and anastomosing immature bone trabeculae intermixed with scattered clusters of multinucleated giant cells. We hereby report a case of a rare hybrid lesion comprising CGCG and JTOF.

## Introduction

Central giant cell granuloma (CGCG) is believed to be an entity exclusive to jaw bones ^1^. It occurs anteriorly in either jaw but is more commonly encountered in the mandible. The majority of cases manifest in children or young adults and are twice as frequent in females than males ^2, 3^. It attains a quite benign behavior, although rare aggressive lesions have been documented ^3^.

The term “benign fibro-osseous lesions” generically describes an aggregate of pathologies that are characterized by replacement of normal bone with a highly cellular connective tissue associated with newly formed bone trabeculae ^4^. These lesions encompass varying entities including neoplastic, dysplastic and reactive pathologic processes ^1^. Ossifying fibroma (OF) and its subtypes represent their neoplastic entity. Juvenile OF (JOF) is an aggressive subtype of ossifying fibromas that can be further classified into juvenile trabecular OF (JTOF) and juvenile psammomatoid OF ^5^. JTOF occurs in children and adolescents with equal distribution of cases among both sexes. It affects the maxilla more than mandible, while other skeletal bones are rare to be affected with such lesion ^3^.

Radiographically, CGCGs usually present as well-demarcated radiolucencies that are multilocular, although unilocular lesions have been noted ^6^. As for JOFs, despite their non-encapsulated nature, they appear well delineated, with early lesions found as unilocular radiolucencies. However, old lesions more commonly assume the multilocular configuration ^7^. Depending on the degree of ossification within the lesion, JOF can present varying extents of radioopacities within its radiographic picture. We can then clarify that those precedent features often challenge the diagnosis and management of such lesions. We hereby report a case of a hybrid lesion that contained both of the previously mentioned entities.

## Case report

**Figure 1.**
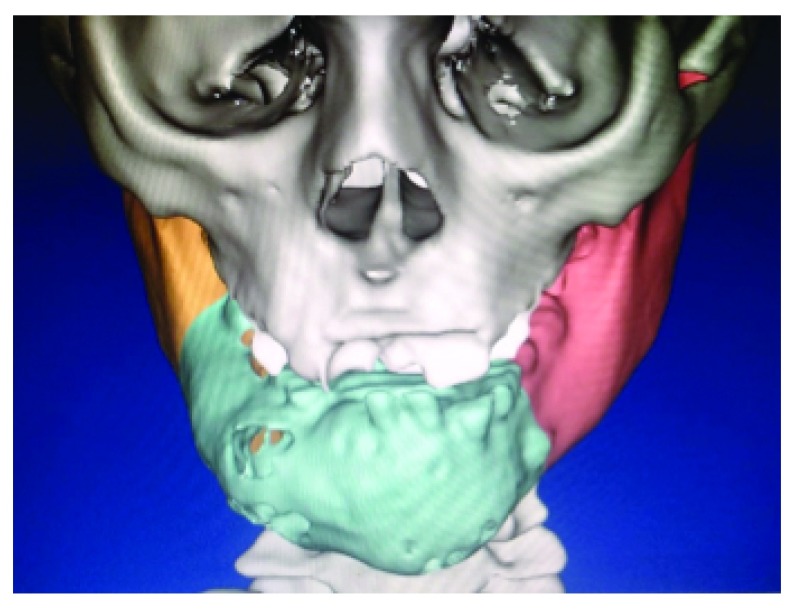
Preoperative Surgical Plan. Preoperative surgical plan showing the swelling in the anterior area of the mandible, causing buccal and lingual bone expansion and displacement of the incisors.

**Figure 2.**
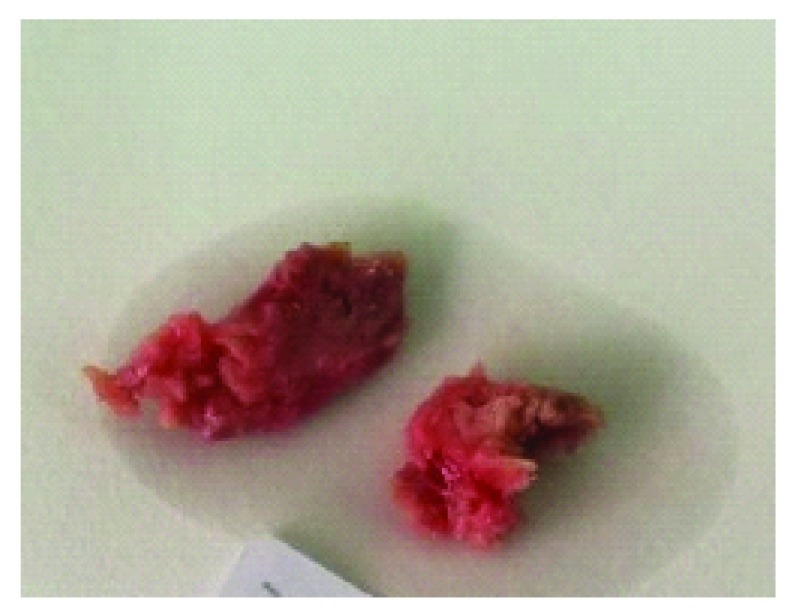
Gross examination of the incisional biopsy. Gross examination of the incisional biopsy revealed two firm pieces of 2.5×2×1.2 cm in size; they were reddish white in color and solid in consistency.

A nine-year-old female patient was reported to the Oral and Maxillofacial Surgery Department at the Faculty of Dentistry, Cairo University, complaining of a painless slowly growing swelling in the anterior area of the mandible of one year’s duration, causing buccal and lingual bone expansion, displacement and looseness of the incisors ( Figure 1). Her medical and family histories were unremarkable. An incisional biopsy was performed, and gross examination revealed two firm pieces 2.5×2×1.2 cm in size, which were reddish white in color and solid in consistency ( Figure 2).

**Figure 3.**
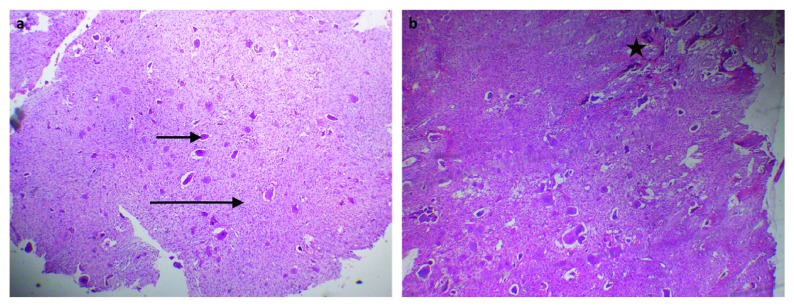
Photomicrograph of old incisional biopsy. ( **a**) Photomicrograph of old incisional biopsy showing heavily scattered multinucleated giant cells (short arrow) in a background of highly cellular fibrous stroma consisting of mononuclear stromal cells (long arrow) and extravasated red blood cells (original magnification 40×). ( **b**) Newly formed bone trabeculae and osteoid tissue (star) were noted at the periphery of the lesion (original magnification 40×)

The histopathological examination using routine H&E staining demonstrated heavily scattered multinucleated giant cells in a background of highly cellular fibrous stroma consisting of mononuclear stromal cells and extravasated red blood cells. Newly formed bone trabeculae and osteoid tissue were noted at the periphery of the lesion ( Figure 3). Accordingly, it was diagnosed as central giant cell granuloma. The surgeon consequently took the decision to inject the lesion with the corticosteroid triamcinolone acetonide (10 mg/ml) dissolved in lidocaine anesthetic solution in equal parts. It was administered in a dosage of 1 ml for every 1 cm ^3^ of the lesion through 6 weekly injections, upon which the lesion showed marked regression.

**Figure 4.**
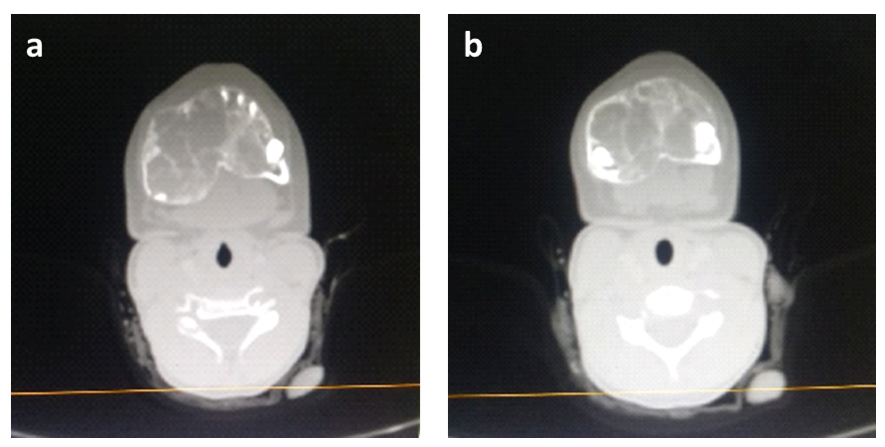
Computed tomography (CT) of the lesion. ( **a**) CT of the lesion showing a well-defined multilocular radiolucency with diffuse flecks of radiopacities, extending from the right impacted third molar area to the left first molar area. ( **b**) CT of the lesion showing perforations in the buccal and lingual cortical plates.

Six months later, the swelling significantly increased in size, causing severe facial disfigurement. Intra-orally, it developed a brownish red discoloration and was non-tender but displaced the otherwise clinically sound related teeth. Computed tomography (CT) of the lesion showed it to be a well-defined multilocular radiolucency with diffuse flecks of radioopacities. It extended from the right impacted third molar area to the left first molar area causing perforations in the buccal and lingual cortical plates ( Figure 4).

**Figure 5.**
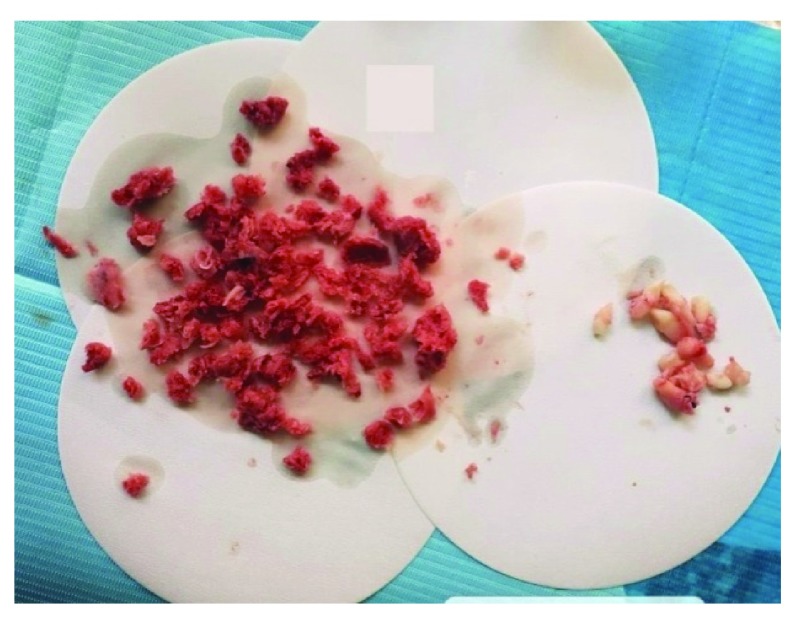
Gross examination of the excised lesion. Gross examination of the excised lesion showing numerous, reddish, hard and soft pieces of tissue that ranged in size, having the average of 2×2×1.5 cm.

**Figure 6.**
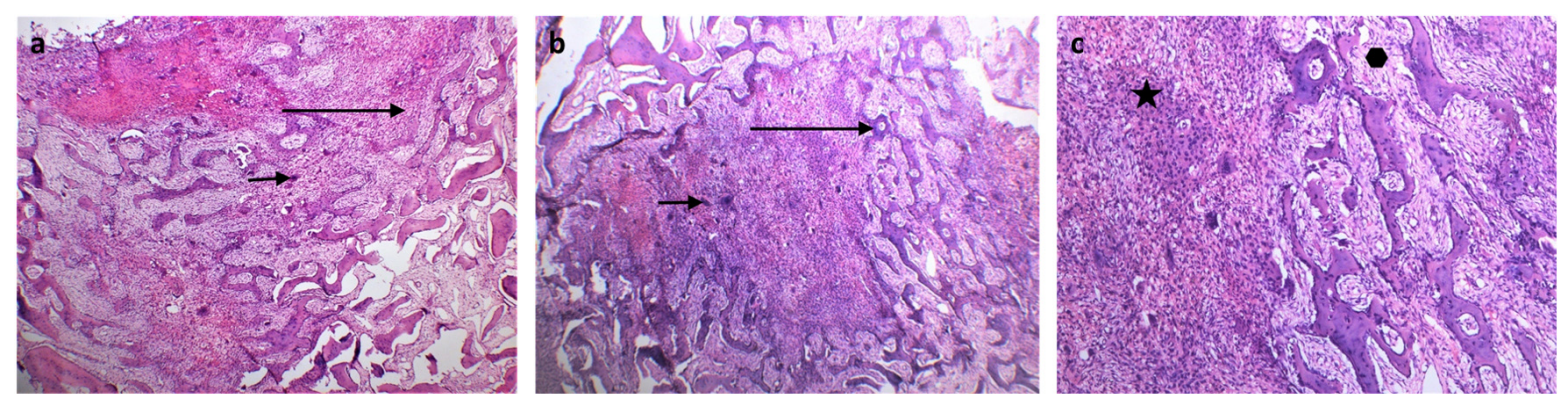
Photomicrograph of the excisional biopsy. ( **a**) Photomicrograph of the excisional biopsy revealed extensive areas of cell-rich connective tissue stroma containing bands of osteoid matrix and anastomosing immature bone trabeculae (long arrows) (original magnification 40×). ( **b**) Scattered clusters of multinucleated giant cells (short arrows) (original magnification 40×). ( **c**) Plump osteoblasts can be seen surrounding the interconnecting immature bony trabeculae. Cellular osteoid (star) and some myxomatous areas (polygon) can also be detected (H&E stain, magnification 100×).

The lesion underwent an excisional biopsy. On gross examination, it consisted of almost 30 pieces that ranged in size, with an average size of 2×2×1.5 cm. They were reddish in color, some were hard and others were soft in consistency ( Figure 5). The specimen also contained multiple tooth follicles of both the primary and permanent dentitions. Sections of this biopsy revealed a highly cellular fibrous connective tissue encompassing numerous multinucleated giant cells. Areas of intralesional hemorrhage were also evident; two findings that are consistent with the diagnosis of central giant cell granuloma. However, other sections revealed extensive areas of cell-rich connective tissue stroma containing bands of osteoid matrix and anastomosing immature bone trabeculae surrounded by plump osteoblasts intermixed with scattered clusters of multinucleated giant cells ( Figure 6), features that are characteristic of juvenile trabecular ossifying fibroma. We subsequently reached the final diagnosis of a hybrid lesion demonstrating central giant cell granuloma in occurrence with juvenile trabecular ossifying fibroma.

Following treatment, the patient was administered 50 mg diclofenac potassium tablets through oral administration, three times daily for one week. The patient responded well to both the surgery and post-operative treatment. The healing was uneventful and no worrying findings were observed through the 6-week follow up.

## Discussion

Central giant cell granuloma (CGCG) is a benign intra-bony lesion that was first introduced to the medical literature by Jaffé in 1953 ^8, 9^. It is a pathology that more commonly affects young females. Some authors like Chuong *et al*. ^10^ and Ficarra *et al*. ^11^ tend to classify it into aggressive and non-aggressive subcategories, due to varying clinical courses of reported cases ^12^. Similarly, it can represent varying radiological pictures ranging from undulated unilocular to multilocular radiolucencies ^13^.

JOF is an aggressive, non-odontogenic, benign tumor of the bone ^14^. It is an entity that affects individuals younger than 15 years of age with no sex predilection ^15^. Radiographically, it is shown as a well-circumscribed unilocular or multilocular radiolucency that may exhibit ground-glass or even more discrete opacifications ^7, 16^.

The term “hybrid lesions” was first introduced by Waldron and El-Mofty during their work on cases of ameloblastoma ^17^. Later, it was generally accepted that “hybrid” lesions represent those lesions that comprise significant portions of histopathological features of multiple, completely different entities ^18– 20^. We believe that this reported case represents a hybrid lesion. Previous cases of hybrid CGCGs associated with different fibro-osseous entities have been reported in the literature. Until now, to our knowledge ten cases of hybrid lesions have been documented. Out of them, five cases were of CGCG associated with ossifying fibroma, three cases of CGCG with fibrous dysplasia and one case was of CGCG with cemento-osseous dysplasia ^21^. To our knowledge, excluding this case, only one analogous case has been reported ^22^.

As regards to the histopathological picture of the two lesions, In some instances, CGCG can show confined peripheral areas of reactive bone and osteoid formation in its highly vascular and cellular connective tissue stroma, as indicated by De Corso *et al*. ^9, 23^ Moreover, in juvenile ossifying fibroma, multinucleated giant cells and areas of hemorrhage can be found scattered in its sometimes hypercellular fibrous matrix ^7^, characteristics that can undoubtedly confuse investigators upon making a diagnosis and accordingly, choosing a treatment plan. An additional fact that may further complicate case management is the presence of hybrid lesions.

The association of giant cells with fibro-osseous lesions could be explained on the basis of different postulations made by many authors. In the case report of Shetty *et al.* ^24^, the authors state it may just be a coincidental finding or correlated by representing a “reactive process rather than a feature of a separate lesion”. While Farzaneh *et al*. ^25^ and Penfold *et al*. ^26^ assume that “osteoblasts may activate osteoclast-type giant cells through paracrine mechanisms” ^27^. Accordingly, a debate has risen between two opinions, whether JOF had developed in a more central position first and then initiated an adjacent giant cell reaction or whether two lesions simply originated independently.

Considering this specified case, we were startled by the prominent divergence in the course of the lesion; i.e., its response to the selected treatment option. It raised multiple questions and so a decision was made to put the intrigue at ease by embarking on research to find answers. It was firstly suspected that there was a concealment of some histopathological features in the previous biopsy that could direct us to accurate deductions on such a lesion. This instinct was fortunately confirmed. By returning to the histopathological slides of the previous incisional biopsy, very minimal marginal areas of the lesion did indeed contain areas of fibrous stroma with few immature bony trabeculae, which led to the thought that they could in fact be indicative of juvenile ossifying fibroma, of the trabecular subtype, rather than what was previously thought to have been regions of an inflammatory bodily reaction in the form of ossification. Bearing that deduction in mind, along with the results of the recent biopsy, and correlating this to the clinico-radiographical information obtained, the case was identified as a hybrid lesion of both CGCG and JTOF.

Owing to the small number of reported hybrid lesions, their biologic behavior cannot be predicted and hence their treatment is uncertain. As for the chosen treatment in our case, no other surgical intervention was made in an attempt by the surgeon to be as conservative as possible considering the young age of the patient. The surgeon also refrained to prescribe any pharmacological agents with the exception of analgesics three times daily for one week after surgery. The patient was given the appropriate self-care instructions following surgery in front of her guardian and the latter was asked to report the patient to the operating surgeon for follow up. The healing was uneventful for six weeks, but the patient was unfortunately lost for the planned three, six and twelve-month follow up.

To conclude, in our search of the literature no cases of similar hybrid lesions were reported, except for one reported by Geetha *et al*. ^22^ To our knowledge, our case is the second to be documented as a hybrid lesion involving CGCG and JOF. Therefore, we believe that this report would significantly contribute to the understanding and clinical management of such a rare lesion.

## Data availability

### Underlying data

All data underlying the results are available as part of the article and no additional source data are required.

## Consent

Written informed consent for publication of clinical details and clinical images was obtained from the guardian of the patient.
