# Supplementary material for: Case Report: rare hybrid lesion of a central giant cell granuloma within a juvenile ossifying fibroma
Source: F1000Res. 2019 Jul 30;8:1218. [Version 1] doi: 10.12688/f1000research.19891.1 (PMC6784877; doi:10.12688/f1000research.19891.1)
Supplement: Peer review history

# Peer review: Reviewer response for version 1

**DOI:** 10.5256/f1000research.21825.r54099

**Published:** 2019-10-07

**Authors:** Rania H Younis

**Affiliations:** 1 School of Dentistry, University of Maryland, Baltimore, MD, USA

(JTOF) The authors present a hybrid case report of CGCG, with juvenile trabecular ossifying fibroma.

 The authors should mention the soft ware used to do the surgical planning (Figure 1), was it a CBCT?

 It is recommended that the authors provide a high power representative of the heavily cellular stroma and pseudoaneurysmal spaces, characteristic of the Juvenile trabecular ossifying fibroma (JTOF).

 Add reference to support the triamcinolone acetonide injection.

 In the discussion the authors need to reflect more on the differential diagnoses, that the bone deposition may be part of a reparative granuloma histologically, yet the rapid clinical expansion speaks against a reparative process.

 That JTOF can be associated with MNGCs, yet the incisional biopsy had enough evidence of a CGCG component to support a hybrid lesion.

I confirm that I have read this submission and believe that I have an appropriate level of expertise to confirm that it is of an acceptable scientific standard, however I have significant reservations, as outlined above.

## References

[1] : Juvenile Trabecular Ossifying Fibroma. *Head Neck Pathol*.2018;12(4) : 10.1007/s12105-017-0862-6 567-571 10.1007/s12105-017-0862-6 29027609PMC6232201

# Peer review: Reviewer response for version 1

**DOI:** 10.5256/f1000research.21825.r52552

**Published:** 2019-09-18

**Authors:** Suwarna B. Dangore-Khasbage

**Affiliations:** 1 Sharad Pawar Dental College and Hospital, Datta Meghe Institute of Medical Sciences (Deemed University), Wardha, Maharashtra, India

Authors have described an uncommon case of  Introduction is relevant, adequate and properly written. a  case report is described in proper sequence with investigation and diagnosis with justification. Enough details are provided about the case. 

 Sufficient discussion is included depicting the importance of the findings and their relevance to future understanding of disease processes

 The overall case report is interesting and useful to readers.

I confirm that I have read this submission and believe that I have an appropriate level of expertise to confirm that it is of an acceptable scientific standard.

# Peer review: Reviewer response for version 1

**DOI:** 10.5256/f1000research.21825.r51869

**Published:** 2019-08-15

**Authors:** Maedeh Ghorbanpour

**Affiliations:** 1 Department of Oral and Maxillofacial Pathology, Faculty of Dentistry, Tehran Medical Sciences, Islamic Azad University, Tehran, Iran

1. In keywords: Fibrous dysplasia should be deleted.
2. Please check the keywords to be according to MeSH system.
3. In introduction: Last sentence of second paragraph about JTOF? In most references psammomatoid type of the JOF is more common in craniofacial skeleton than trabecular type
4. Figure 1: It would be better to add panoramic view or CBCT of the lesion.
5. Figure 6C: It would be better to use higher magnifications for showing osteoblasts and cellular osteoid. 
6. The first sentence of the “Discussion” needs appropriate citation. The original citation needs to be applied to address Jaffé, 1953 original article.
7. Is this case the first hybrid case of CGCG and JTOF? If not, please provide a literature review of the same cases in a table.
8. It is recommended to summarize previously reported cases of hybrid CGCG in a separate table.
9. The manuscript needs **major revision**.

I confirm that I have read this submission and believe that I have an appropriate level of expertise to confirm that it is of an acceptable scientific standard, however I have significant reservations, as outlined above.
